# Hospital Processes and the Nurse-Patient Interaction in Breast Cancer Care. Findings from a Cross-Sectional Study

**DOI:** 10.3390/ijerph18158224

**Published:** 2021-08-03

**Authors:** Johanna Sophie Lubasch, Susan Lee, Christoph Kowalski, Marina Beckmann, Holger Pfaff, Lena Ansmann

**Affiliations:** 1 Organizational Health Services Research, School of Medicine and Health Sciences, Carl von Ossietzky University of Oldenburg, Ammerlaender Heerstr. 140, 26129 Oldenburg, Germany; lena.ansmann@uol.de; 2 Institute of Medical Sociology, Health Services Research, and Rehabilitation Science (IMVR), Faculty of Human Sciences, Faculty of Medicine and University Hospital Cologne, University of Cologne, Eupener Str. 129, 50933 Cologne, Germany; susan.lee@uk-koeln.de (S.L.); marina.beckmann@uk-koeln.de (M.B.); holger.pfaff@uk-koeln.de (H.P.); 3 Department Accreditation, German Cancer Society, 14057 Berlin, Germany; kowalski@krebsgesellschaft.de

**Keywords:** multilevel analysis, patient-nurse relation, organizations, social support, process organization

## Abstract

(1) Background: Evidence suggests that organizational processes of hospitals have an impact on patient-professional interactions. Within the nurse-patient interaction, nurses play a key role providing social support. Factors influencing the nurse-patient interaction have seldomly been researched. We aimed to examine whether the process organization in hospitals is associated with breast cancer patients’ perceived social support from nurses.; (2) Methods: Data analysis based on a cross-sectional patient survey (2979 breast cancer patients, 83 German hospitals) and information on hospital structures. Associations between process organization and perceived social support were analyzed with logistic hierarchical regression models adjusted for patient characteristics and hospital structures.; (3) Results: Most patients were 40–69 years old and classified with UICC stage II or III. Native language, age and hospital ownership status showed significant associations to the perception of social support. Patients treated in hospitals with better process organization at admission (OR 3.61; 95%-CI 1.67, 7.78) and during the hospital stay (OR 2.11; 95%-CI 1.04; 4.29) perceived significantly more social support from nurses.; (4) Conclusions: Designing a supportive nursing work environment and improving process organization in hospitals may create conditions conducive for a supportive patient-nurse interaction. More research is needed to better understand mechanisms behind the associations found.

## 1. Introduction

Among women, breast cancer is the most frequent cancer worldwide, with 2.1 million women affected each year [1]. In Germany, about 70,000 women receive a breast cancer diagnosis annually [2]. German practitioner guidelines recommend for breast cancer patients to be treated in accredited breast cancer centers [3] in which multidisciplinary experts work closely together and recommend individual therapy plans in multidisciplinary tumor conferences to guarantee the best possible therapy [3]. In Germany, the quality of breast cancer centers is assured annually by independent institutions [3,4].

Receiving a breast cancer diagnosis often entails physical and psychosocial stressors [5]. To handle these stressors, breast cancer patients need to be supported emotionally and socially. Social support thereby fosters the persons’ ability to cope with and adjust to the disease [6]. It has an impact on various health outcomes, e.g., quality of life and depression [7,8,9] and was found to be associated with various patient outcomes in cancer, e.g., higher self-efficacy as well as higher quality of life [10,11,12]. 

### 1.1. Importance of Social Support for Breast Cancer Patients

Social support is defined as support in emotional, informational and instrumental form contributing to assist a person in a burdensome situation [13]. Besides family members and friends [6,12], healthcare professionals are regarded by patients as important sources of social support [12]. Healthcare professionals provide social support within trustful patient-professional interactions in terms of praise, motivation, encouragement, reassurance, advice and advocacy [14]. Hereby and especially in cancer care nurses play a key role since they are often accompanying patients throughout the time from diagnosis to treatment, or during palliative care [15,16]. High quality nurse-patient interactions have been found to have a positive impact on recovery times and physical as well as psychological morbidity and mortality [17].

### 1.2. Association between Organizational Factors and Patient-Provider Interactions

According to the Institute of Medicine, each level in the health care delivery system affects the level(s) below (for example, the hospital environment affects individual nurses and patients) [18]. In the hospital context, environmental factors having an impact on patient care are e.g., size, ownership status, teaching status or nurse staffing [19,20,21]. Research on associations between the patient-professional interaction and environmental factors has predominantly focused on the patient-physician interaction and rarely on the patient-nurse interaction. Studies revealed that the social support provided by physicians is associated with the work environment (e.g., social capital) or workload [22,23]. Furthermore, an association between the interaction of patients and physicians and the hospital’s process organization (e.g., coordination between wards or communication between nurses and physicians) was shown [24]. Concerning the interaction between patients’ and nurses’ studies revealed correlations with working climate, nurses’ lack of time and a high work load [25,26]. To our knowledge the influence of process organization on the patient-nurse interaction is mostly unexplored. However, communication models for cancer nursing point out that communication with cancer patients needs to be prepared and take place in a quiet and unstressed environment where time constraints are hindering [27]. We therefore assume that in hospitals having problems with organizational processes, the nurse-patient interaction might be adversely affected with regard to the provision of social support. A better understanding of these associations is important to elaborate measures improving the nurse work environment and fostering the provision of social support as part of the patient-nurse interaction as well as the associated positive effects of social support on patients.

**Figure 1:**
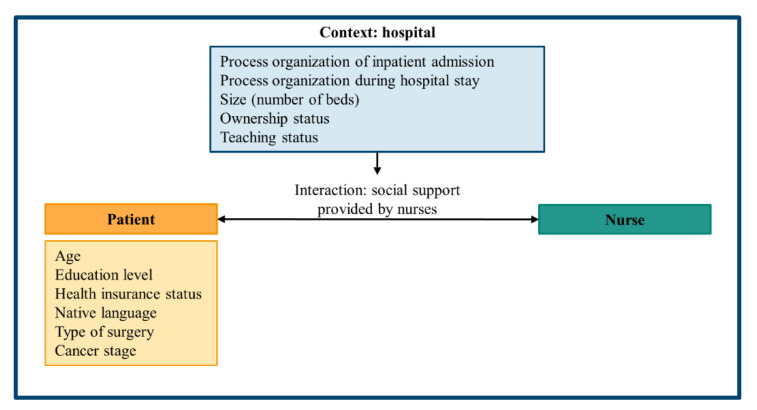
Research model: patient-nurses interaction within contextual factors.

For our study, we developed a research model based on the patient–professional communication framework by Feldman-Stewart et al. [28]. The framework inter alia emphasizes that the patient-professional interaction and its outcomes are indirectly affected by a complex environment of factors outside the communication process. The impact of contextual factors on the communication process, however, is not yet strongly illuminated by the framework. In order to shed light on this, we hypothesize that problems with process organization are an environmental factor having an impact on communication processes and therewith on the provision of social support. Therefore, the study’s research question was as follows (see Figure 1 for research model):

Is the process organization of hospital care associated with social support from nurses provided in breast cancer care?

## 2. Materials and Methods

In a secondary data analysis, data from two sources were combined: a cross-sectional patient survey in breast cancer center hospitals and structured quality reports of the same hospitals.

### 2.1. Patient Survey

#### 2.1.1. Data Collection

Survey data was collected in 2013 in 83 hospitals accredited as breast cancer centers in the German federal state of North Rhine-Westphalia. Before discharge, patients were asked to give written consent to participate in the survey. If they agreed, a postal questionnaire was sent to their address, and the hospital personnel provided additional data on disease and therapy-related characteristics. According to Dillman’s Total Design Method, three contact attempts were made per patient [29]. More details on the survey can be found elsewhere [4,24].

#### 2.1.2. Sample

Patients were included if they (1) were older than 18 years, (2) had undergone inpatient surgery between 1 February and 31 July 2013, for newly diagnosed breast cancer, (3) had at least one malignancy, and (4) had at least one postoperative histological evaluation. Of 5583 patients who were asked to participate in the defined period, 4841 consented to participate in the study and 4217 returned completed questionnaires (response rate: 75.5%). 

#### 2.1.3. Instruments

The survey was conducted using the Cologne Patient Questionnaire for Breast Cancer (CPQ-BC) which consists of various validated instruments and self-developed instruments. It is used annually in a cross-sectional survey in hospitals accredited as breast cancer and has been shown to have good psychometric properties [4]. The scales used in the present analysis have all been self-developed.

The dependent variable, patients’ perceptions of social support from nurses, was measured the SuPP-N scale (validation not yet published) which consists of three items (Cronbach’s alpha = 0.91) (displayed in Table 1). The process organization of inpatient admission (Cronbach’s alpha = 0.81) was measured with four items and process organization during the hospital stay (Cronbach’s alpha = 0.87) was measured with six items (items see Table 1).

All items of the three scales were rated using four response options, ranging from “strongly disagree” to “strongly agree”. The negatively phrased items of the scale measuring process organization during the hospital stay were recoded, so that higher values indicate better processes. A score was calculated for each of the scales by summing the item responses and dividing the sum by the number of items. Higher values indicated more social support and better process organization, respectively. Due to its highly skewed distribution, the variable “perceived support from nurses” was dichotomized [30]. Dichotomization was achieved by combining the three highest quarters (>3.33), indicating high support, and comparing this category with the lowest quarter (≤3.33), indicating less than high support. We were thereby able to differentiate patients who perceived the highest support from those who perceived less than high support.

The following variables were used to control for case-mix differences: age (in categories), educational level (without lower secondary school education, lower secondary school education, intermediate secondary school education, university entrance qualification), health insurance status (public insurance, public with supplementary private insurance, private insurance), native language (German, other), type of surgery (mastectomy with reconstruction, mastectomy without reconstruction, breast conserving therapy), physical status (classified according to the American Society of Anesthesiologists [ASA]) and cancer stage (classified according to the Union for International Cancer Control [UICC] staging system).

### 2.2. Structured Quality Reports of the Hospitals

To control for hospital structures, on the hospital level number of beds (as indicator of hospital size), ownership status and teaching status were used. In Germany, hospitals are legally obliged to publish annual structured quality reports, which provide an overview of hospital services and structures. From these quality reports, the information on hospital structures was extracted. Ownership status comprised the three categories “for-profit”, “charitable”, and “public”. Teaching status comprised the three categories “non-teaching hospital”, “academic teaching hospital” and “university hospital”. Number of beds was added to our regression as a continuous variable.

### 2.3. Data Analysis

The research question of whether the process organization of inpatient care is associated with social support from nurses was analyzed by conducting hierarchical logistic regression. Male participants were excluded from the analysis because of their small number (*n* = 26). Due to missing values on continuous variables, the analysis is based on a sample of 2979 patients. For categorical variables, missing data were included in the model as separate dummy variables to avoid case deletion. No imputations were performed. All independent variables have been tested for multicollinearity. Taking into account the clustered structure of the data (patients nested in hospitals), data were analyzed using stepwise two-level random intercept hierarchical logistic regression models with restricted maximum likelihood estimation [31]. In model 1, patient characteristics were included on the individual level. In model 2, hospital structures were added on the hospital level. In the third model, the two process variables were aggregated and included on the hospital level. To control for interindividual differences, the individually measured process variables were additionally included on the individual level, as described in contextual analysis models [32]. To determine the proportion of variance in perceived support that is attributable to the hospital level, we calculated intraclass correlation coefficients (ICC) and R² for all models. IBM^®^ SPSS^®^ 26.0 (IBM Corporation, Armonk, NY, USA) was used for descriptive analysis and MPlus Version 8.2 (Muthen & Muthen, Los Angeles, CA, USA) for multilevel analysis. As significance level α = 0.05 was chosen.

### 2.4. Ethics and Other Permission

Consultation by and positive votes from the Ethics Committee of the Faculty of Medicine of the University of Cologne was obtained prior to the start of the study (number: 06–010). All participants provide written consent.

## 3. Results

### 3.1. Descriptive Results

The participants predominantly reported to have received social support by nurses and organizational processes were reported to be good (see Table 1). Most participants in the sample were classified with UICC stage I (39.4%) or stage II (26.2%) (see Table 2). Most patients had undergone breast conserving therapy (72.7%). The majority of participants was classified as ASA 1 or 2 (48.1%; 37.1%). Mean age of participants was 58.5 years, and most participants declared German as their native language (93.8%). 

According to the structured quality reports, most of the hospitals were teaching hospitals (83.1%), (see Table 3). Moreover, most hospitals were in charitable ownership (72.3%). The smallest hospital provided 43 patient beds and the largest 1422 patient beds (average = 526 beds).

### 3.2. Results of Multilevel Analysis

The independent variables showed no multicollinearity (results not presented). The null model revealed an ICC of 0.021, indicating that 2.1% of the variance in perceived support from nurses can be attributed to the hospital level (see Table 4). 

On the patient level, age was consistently associated with social support (see Table 4). Patients aged 60 to 69 reported significantly more social support than patients aged 50 to 59. Moreover, patients with university entrance qualification reported significantly less social support from their nurses compared to patients with a lower secondary school leaving certificate (OR 0.85; 95% CI 0.73, 0.99). However, the association did not remain significant in model 2 or model 3. In model 3, patients indicating German as their native language reported more social support than did patients with another native language (OR 1.32; 95% CI 1.01, 1.72), and patients with public health insurance reported significantly more social support than did patients with private health insurance (OR 1.29; 95% CI 1.02, 1.62).

On the hospital level, in model 3 the process organization of inpatient admission and the process organization during hospital stay were significantly positively associated with perceived social support from nurses (see Table 4). Additionally, the model showed a significant positive relationship between for-profit ownership and support from nurses, compared to public ownership (OR 1.41; 95% CI 1.02, 1.93). Moreover, the final model 3 showed the largest reduction in unexplained variance on the hospital level (57.1%).

## 4. Discussion

To our knowledge, this study is the first to determine associations between hospitals’ process organization and the nurse-patient interaction in terms of social support. The results of the multilevel models reveal patients felt more supported by nurses in hospitals in which patients, on average, experienced the inpatient admission and the hospital stay to be better organized. 

### 4.1. Interpretation within the Context of the Wider Literature

The results of the study align with results of previous studies, which showed associations between process organization and the provision of social support from physicians in breast cancer care [22,24]. The results are also in line with the communication framework developed by Feldman-Stewart et al. [28], which suggests patient-professional interactions to be influenced by the context in which it takes place. Furthermore, the results supplement findings from previous literature in the nursing field, showing that the nurse-patient interaction is shaped by its environment e.g., in terms of workplace culture [20]. However, neither our study nor the model can provide detailed explanations for the observed associations between process organization and social support from nurses. We assume that nurses working in hospitals having problems with process organization might have less time or capacity to interact with their patients because they are preoccupied with managing these processes as already assumed by Ansmann et al. [24] concerning social support provided by physicians. Considering the shortage of healthcare professionals in German hospitals, we further assume that deficits in process organization reflect stress and high workload among nurses. High workload among nurses, in turn, has been found to be negatively associated with the nurse-patient interaction [25], which supports our assumption of stress impacting the provision of less social support. 

Comparing the R² of model 2 and model 3 reveals that the addition of the two process organization variables in model 3 leads to a more substantial decrease of ICC than did the addition of hospital structures in model 2. This suggests that processes may be more important determinants of social support from nurses than hospital structures. 

Concerning patient characteristics, associations were found between age, native language as well as health insurance status on the one hand and perceived social support from nurses on the other. The finding that patients aged 60–69 years perceived more social support from nurses than 50–59-year-old patients confirms results from Puts et al. [33], who revealed that younger age of cancer patients is associated to more reported unmet psychosocial, informational and physical needs. They discuss that older patients might be unaware of supportive care interventions or might have lower health literacy, which in turn might be associated with less supportive care needs. However, in our study, the significant association between age and social support could only be observed in one age category and should not be generalized. The results showing that patients with German as their native language reported more social support from nurses’ supplement results from Kowalski et al. [34], who found out that patients with German as their native language are more likely to be satisfied with the nurse staff. An intuitive explanation for this is that language barriers affect the nurse-patient interaction and thus the provision of social support. Furthermore, our results show an association between public health insurance status and higher levels of perceived social support. We suspect that patients with public health insurance may be less demanding or critical and therefore tend to report more social support. However, native language and insurance status only show significant results in the third model, suggesting that the associations can be ascribed to the high variance explanation in this model.

Concerning hospital structures, significant results were found for ownership status. In hospitals with for-profit ownership, patients reported significantly more social support from nurses than in public hospitals. However, on the basis of our data, we cannot provide explanations for this result, and results from previous studies concerning associations between hospital ownership and patient care have been inconsistent [23,35]. The fact that significant results are found for ownership only in the third model might be ascribed to the high variance explanation achieved by adding the variables describing process organization. Teaching status and number of beds did not show any significant association with the perception of social support from nurses.

### 4.2. Strengths and Limitations 

A strength of our study is the high number of participating patients and hospitals and the high response rate. Moreover, to control for the interdependence of observations of patients receiving care within the same hospital, multilevel analyses were conducted [31], which has emerged as the method of choice in considering contextual factors in health services research [36]. In addition, a number of patient and hospital characteristics that have previously been found to influence care outcomes were extensively controlled within the models. However, like any cross-sectional study, this study is not suitable for examining causality. Moreover, we conducted the study in only one federal state in Germany, although the most populous state with 20% of the total breast cancer incidence occurring here. Unfortunately, we were not able to perform a non-responder analysis. However, the average age and the distribution of UICC stages of our participants are comparable to the age and UICC stages of women with newly diagnosed breast cancer in Germany [2]. Moreover, the ICC for the null model was relatively low (0.021). However, similarly low variance between standardized healthcare organizations such as accredited cancer centers has been found in many previous studies in health services research [22,37]. Ben Charif et al. [37] compared ICC values for shared decision-making measures in primary care and revealed values between 0.02 and 0.06. Moreover, Selby et al. [38] observed that quality improvements at healthcare facilities have generated clinically significant improvements, although ICC levels were low from the beginning. We furthermore assume, that the provision of social support might show higher variations between the wards, which do not become apparent when comparing on hospital level. However, the data did not allow an analysis of the ward-specific variation. Our study is also at risk of common method bias because some predictor variables and the outcome measure were both reported in the same patient survey. However, consideration of the patient’s perspective is important for gaining insights into aspects of healthcare which cannot be measured objectively [39]. We further suggest that common method bias might have been reduced by aggregating the data and analyzing it on the hospital level rather than on the individual level, on which it was originally measured. Moreover, we are aware that the secondary data from 2013 probably does not reflect recent trends in healthcare. However, we believe that the associations found only vary by time regarding their strength and are rather basic and stable relationships.

### 4.3. Implications for Policy, Practice and Research

The findings of our study suggest that investing in better-organized processes in hospitals may facilitate a supportive nurse-patient interaction in breast cancer care. To address this, health policy and hospital management should strive to create conditions to optimize processes in hospitals in a patient-centered way. Possible actions to reach this goal could be the implementation of standardized work processes or the restructuring of workplaces to foster well-organized and effective work processes as suggested before in the context of hospital discharge [40]. Considering that deficits in process organization might cause stress and higher workload among nurses and other professions, hospital managers’ actions should moreover focus on designing a supportive work environment for nurses. This may be even more important in view of the recent shortages of healthcare professionals in hospitals in Germany and many other countries. In this context, it was previously suggested to apply a moderated group procedure in with unit managers and registered nurses and if necessary, representatives of other professions in order to identify deficits in work organization and develop, implement and test possible improvement measures [41]. However, before being able to give concrete recommendations for practice implications, studies should be conducted to better understand the mechanisms behind the association between process organization and the patient-nurse interaction. Moreover, patients and nurses from the same healthcare organizations could be surveyed to combine data from the patients’ perspective with data from the nurses’ perspective in order to gain deeper insights into possible barriers and facilitators of the patient-nurse interaction.

## 5. Conclusions

This study provides preliminary evidence that the social support breast cancer patients receive from hospital nurses is affected by the process organization within the hospital. The results indicate that improving hospital processes may be conducive for a supportive patient-nurse interaction and therewith may improve patient outcomes. More research is needed to better understand mechanisms behind the associations and to give concrete recommendations for action.

## Figures and Tables

**Table 1:** Items of the scales measuring social support provided by nurses (SuPP-N) and measuring process organization.

		Response Options: Frequency (%) ^a^ (*n* = 2979)			
Item	Item Content	1	2	3	4
**SuPP-N scale**					
suppn1	I could rely on the nurses when I had problems with my illness.	15 (0.5)	90 (3.0)	625 (21.0)	2249 (75.5)
suppn2	The nurses supported me in a way that made it easier for me to deal with my illness.	21 (0.7)	125 (4.2)	707 (23.7)	2126 (71.4)
suppn3	The nurses were willing to listen to my illness-related problems.	38 (1.3)	175 (5.9)	787 (26.4)	1979 (66.4)
**Process Organization of Inpatient Admission**					
1	The patient admission was easy to find.	15 (0.5)	75 (2.5)	575 (19.3)	2314 (77.7)
2	The waiting time at the admission was short.	90 (3.0)	281 (9.4)	807 (27.1)	1801 (60.5)
3	The admission forms were understandable.	13 (0.4)	54 (1.8)	722 (24.2)	2190 (73.5)
4	The admission process was quick.	50 (1.7)	159 (5.3)	660 (22.2)	2110 (70.8)
**Process Organization during the Hospital Stay**					
1	On the day of my admission there were organizational problems.	2085 (70.0)	516 (17.3)	249 (8.4)	129 (4.3)
2	Here at the hospital, the right hand sometimes didn’t know what the left hand was doing.	2059 (69.1)	661 (22.2)	192 (6.4)	67 (2.2)
3	There were often waiting times for the examinations or procedures.	1385 (46.5)	937 (31.5)	516 (17.3)	141 (4.7)
4	Examinations and procedures were sometimes rescheduled.	1918 (64.4)	690 (23.2)	264 (8.9)	107 (3.6)
5	I had the impression, that there were coordination difficulties between the ward and the diagnostical examination units.	2032 (68.2)	678 (22.8)	194 (6.5)	75 (2.5)
6	I had the impression that, there were coordination difficulties between doctors and nurses.	2168 (72.8)	651 (21.9)	112 (3.8)	48 (1.6)

^a^ ”I strongly disagree” (1), “I somewhat disagree” (2), “I somewhat agree” (3), “I strongly agree”(4). Note: Due to rounding, percentages might not add up to exactly 100%.

**Table 2:** Descriptive results of the patient-level variables (*n* = 2979).

Variable	Response Trait	*n* (%)
UICC Staging	0	318 (10.7)
	I	1175 (39.4)
	II	781 (26.2)
	III	235 (7.9)
	IV	99 (3.3)
	Missing	371 (12.5)
Neoadjuvant Chemotherapy	Yes	330 (11.1)
	No	2619 (87.9)
	Missing	30 (1.0)
Type of surgery	Breast-conserving surgery	2166 (72.7)
	Mastectomy	705 (23.6)
	Missing	108 (3.6)
ASA	1	1434 (48.1)
	2	1105 (37.1)
	3 and 4	249 (8.4)
	Missing	191 (6.4)
Age	18–39	122 (4.1)
	40–49	547 (18.4)
	50–59	932 (31.3)
	60–69	810 (27.2)
	70–79	452 (15.2)
	≥80	101 (3.4)
	Missing	15 (0.5)
Highest educational level	Without lower secondary school education	51 (1.7)
	Lower secondary school education	1148 (38.5)
	Intermediate secondary school education	864 (29.0)
	university entrance qualification	849 (28.5)
	Missing	67 (2.2)
Native language	German	2795 (93.8)
	Other	184 (6.2)
	Missing	32 (1.1)
Health insurance status	Public	2087 (70.1)
	Public with additional private insurance	489 (16.4)
	Private	355 (11.9)
	Missing	48 (1.6)
Variable	Scale format	Mean (SD); min-max
Perceived social support	Scale from 1 to 4	3.65 (0.55); 1–4
Process organization of inpatient admission	Scale from 1 to 4	3.63 (0.50); 1–4
Process organization during hospital stay	Scale from 1 to 4	3.50 (0.59); 1–4

UICC staging, cancer stage classified according to the International Union Against Cancer; ASA, physical status classified according to the American Society of Anesthesiologists. Note: Due to rounding, percentages might not add up to exactly 100%.

**Table 3:** Descriptive results of the hospital-level variables (*n* = 83).

Variable	Response Trait	*n* (%)
Teaching status	Non-teaching hospital	14 (16.9)
	Academic teaching hospital	64 (77.1)
	University hospital	5 (6.0)
Hospital ownership status	For-profit ownership	6 (7.2)
	Public ownership	17 (20.5)
	Charitable ownership	60 (72.3)
	**Minimum/Maximum**	**Mean (SD)**
Hospital size (number of beds)	43/1422	526 (284)

SD, standard deviation. Note: Due to rounding, percentages might not add up to exactly 100%.

**Table 4:** Logistic hierarchical regression models with perceived support from nurses as the dependent variable; odds ratios (OR) and 95% confidence interval (95%-CI).

		Model 1	Model 2	Model 3
**Patient Level**		OR (95%-CI)	OR (95%-CI)	OR (95%-CI)
UICC Staging (ref. Stage 0)	Stage 1	1.05 (0.85; 1.30)	1.05 (0.85; 1.29)	1.02 (0.82; 1.28)
	Stage 2	1.17 (0.94; 1.45)	1.18 (0.95; 1.46)	1.25 (0.98; 1.59)
	Stage 3	1.09 (0.75; 1.57)	1.09 (0.76; 1.57)	1.06 (0.73; 1.56)
	Stage 4	1.17 (0.72; 1.88)	1.16 (0.72; 1.88)	1.09 (0.69; 1.71)
Age (ref. 50–59)	18–39	1.24 (0.83; 1.86)	1.25 (0.84; 1.87)	1.44 (0.95; 2.20)
	40–49	1.07 (0.87; 1.32)	1.07 (0.87; 1.32)	1.10 (0.87; 1.36)
	60–69	**1.25 (1.02, 1.52)**	**1.25 (1.03; 1.52)**	**1.28 (1.03; 1.60)**
	70–79	0.96 (0.75; 1.22)	0.96 (0.75; 1.23)	0.88 (0.67; 1.16)
	Older than 79	1.27 (0.90; 1.81)	1.28 (0.90; 1.82)	1.27 (0.90; 1.79)
Neoadjuvant chemotherapy (ref. no)	yes	0.93 (0.75; 1.15)	0.92 (0.75; 1.14)	0.86 (0.69; 1.08)
Type of surgery (ref. mastectomy without reconstruction)	Mastectomy with reconstruction	0.97 (0.74; 1.27)	0.96 (0.73; 1.26)	0.95 (0.70; 1.30)
	Breast-conserving therapy	1.05 (0.86; 1.29)	1.05 (0.86; 1.28)	1.05 (0.85; 1.31)
ASA classification (ref. ASA 1)	ASA 2	1.08 (0.94; 1.25)	1.08 (0.94; 1.24)	1.10 (0.95; 1.27)
	ASA 3 and 4	0.81 (0.59; 1.11)	0.80 (0.59; 1.10)	0.94 (0.65; 1.37)
Highest educational level (ref. Lower secondary school education)	Without lower secondary school education	0.59 (0.34; 1.00)	0.58 (0.34; 1.00)	0.64 (0.37; 1.13)
	Intermediate secondary school education	0.93 (0.70; 1.08)	0.93 (0.79; 1.08)	0.90 (0.75; 1.08)
	university entrance qualification	**0.85 (0.73; 0.99)**	0.86 (0.74; 1.00)	0.82 (0.98; 1.15)
Native language (ref. other)	German	1.23 (0.96; 1.56)	1.23 (0.96; 1.56)	**1.32 (1.01; 1.72)**
Insurance status (ref. private)	Public	1.22 (1.00; 1.51)	1.22 (1.00; 1.51)	**1.29 (1.02; 1.62)**
	Public with additional private insurance	1.06 (0.81; 1.40)	1.07 (0.81; 1.40)	0.99 (0.74; 1.32)
Process organization of inpatient admission				**2.15 (1.79; 2.58)**
Process organization during hospital stay				**2.81 (2.41; 3.29)**
**Hospital level**			OR (95%-CI)	OR (95%-CI)
Hospital size (number of beds)			1.00 (0.99; 1.00)	1.00 (0.99; 1.00)
Teaching status (ref. non-teaching)	academic educational hospital		1.21 (0.94; 1.56)	1.01 (0.76; 1.34)
	university hospital		0.60 (0.72; 2.80)	1.41 (0.67; 2.97)
Hospital ownership (ref. public)	Charitable		1.08 (0.84; 1.40)	1.01 (0.79; 1.28)
	For-profit		1.31 (0.96; 1.79)	**1.41 (1.02; 1.93)**
Process organization of inpatient admission				**3.61 (1.67; 7.78)**
Process organization during hospital stay				**2.11 (1.04; 4.29)**
ICC (nullmodel = 0.021)		0.021	0.018	0.009
R² Level 2		0.0%	14.3%	57.1%

R² Level 2, percentage of explained between-hospital variance; ICC, intraclass correlation coefficient; OR = odds ratio; CI, confidence interval; UICC staging, cancer stage classified according to the International Union Against Cancer; ASA, physical status classified according to the American Society of Anesthesiologists, **bold text** indicates statistically significant results

## Data Availability

The data presented in this study are available on request from the corresponding author.
